# Mechanosensitive Piezo1 Channels Mediate Diaphragm Fibrosis Induced by Prolonged Mechanical Ventilation

**DOI:** 10.1002/jcsm.70136

**Published:** 2025-12-03

**Authors:** Dan Xie, Yuru Xiao, Xuexin Li, Bowen Sun, Jianguo Feng, Jing Jia, Jie Li, Yun Wang, Fei He, Li Liu

**Affiliations:** ^1^ Department of Anesthesiology, Affiliated Hospital Southwest Medical University Luzhou China; ^2^ Department of Anesthesiology, Affiliated Hospital North Sichuan Medical College Nanchong China; ^3^ Anesthesiology and Critical Care Medicine Key Laboratory of Luzhou Southwest Medical University Luzhou China

**Keywords:** diaphragm fibrosis, mechanical ventilation, Nr4a1, Piezo1

## Abstract

**Background:**

Ventilator‐induced diaphragmatic dysfunction (VIDD) is a major complication in critically ill patients. Prolonged mechanical ventilation (MV) triggers diaphragmatic fibrotic remodelling, but the underlying mechanisms remain unclear. This study investigated the role of the mechanosensitive channel Piezo1 in this process.

**Methods:**

A rat model of MV was established for 6 or 12 h. Diaphragm structure (atrophy and fibrosis) and function (frequency‐contraction curve and fatigue index) were assessed. The roles of Piezo1 were probed using the inhibitor GsMTx4 (a nonspecific mechanosensitive channel inhibitor) and adeno‐associated virus (AAV)–mediated knockdown. Downstream signalling was identified by RNA sequencing (RNA‐seq) and validated with cytosporone‐B (CsnB, a specific agonist of Nr4a1).

**Results:**

Compared with controls, MV for 12 h induced significant diaphragm fibrosis, atrophy and dysfunction, alongside increased Piezo1 expression (mRNA: 2.362 ± 0.429 vs. 0.920 ± 0.363, *p* = 0.0018; protein: 1.098 ± 0.103 vs. 0.676 ± 0.102, *p* = 0.0007). Both GsMTx4 and Piezo1 knockdown alleviated these effects. Knockdown reduced the collagen deposition area by approximately 21% and downregulated key fibrotic markers including fibronectin (0.749 ± 0.118 vs. 1.081 ± 0.117, *p* < 0.0001), collagen 1 (0.703 ± 0.087 vs. 1.155 ± 0.131, *p* < 0.0001), collagen 3 (0.879 ± 0.074 vs. 1.063 ± 0.068, *p* = 0.022) and α‐SMA (0.872 ± 0.657 vs. 1.108 ± 0.078, *p* = 0.0031) compared to the MV12 + shCtrl group. RNA‐seq identified Nr4a1 as a downstream factor (*p* value < 0.009). CsnB treatment increased Nr4a1 expression (1.128 ± 0.113 vs. 0.490 ± 0.084, *p* < 0.0001), mitigating prolonged MV‐induced diaphragm fibrosis and dysfunction but not atrophy (938.1 ± 116.2 vs. 754.7 ± 155.5, *p* = 0.1079).

**Conclusions:**

Piezo1 upregulation is a key mechanism in ventilator‐induced diaphragm fibrosis, potentially mediated through the Akt/Nr4a1 signalling pathway. Targeted inhibition of Piezo1 or activation of Nr4a1 presents a promising therapeutic strategy to prevent fibrosis and preserve diaphragm function.

## 1 Introduction

As a key life support technology, mechanical ventilation (MV) has been widely utilized in intensive care units (ICUs) to effectively maintain the respiratory function of critically ill patients [1, 2]. However, prolonged MV can result in rapid diaphragmatic atrophy and contractile dysfunction, a condition collectively known as ventilator‐induced diaphragmatic dysfunction (VIDD). VIDD poses a substantial clinical challenge, affecting about 60%–80% of patients in the ICU, and is associated with weaning difficulties, extended hospital stays, increased mortality rates and poor long‐term prognosis [3, 4, 5]. VIDD is a multifactorial condition driven by accelerated proteolysis, suppressed protein synthesis, diaphragm fibre damage, inflammatory activation and oxidative stress [4, 6].

Notably, although both COVID‐19 and non‐COVID‐19 patients under MV exhibited significant diaphragm atrophy, COVID‐19 patients showed exacerbated extracellular matrix (ECM) deposition [7]. This finding supports the concept of diaphragm replacement fibrosis, where atrophied muscle fibres are partially replaced by ECM, impairing tissue elasticity and contractile function [8, 9, 10]. Although the clinical correlation between MV and diaphragm fibrosis appears to be established, the underlying mechanisms remain unclear. MV induces the production of TGF‐β1 and upregulates the expression of mesenchymal markers through Lumican [11], and the PI3K‐γ pathway mediates MV‐induced fibrosis and dysfunction in the diaphragm [12].

Emerging evidence implicates mechanical stress as a central driver of fibrosis in multiple organs [13, 14, 15]. Mechanical ventilation suppresses the diaphragm's active contractile function and induces passive cyclic stretching in synchrony with lung inflation and deflation [16]. We therefore hypothesize that prolonged passive cyclic stretch of the diaphragm (rather than active contraction) may contribute to diaphragm injury and fibrotic remodelling. No studies have yet explored whether the passive cyclic stretching of the diaphragm during MV can trigger fibrotic signalling through specific mechanosensitive molecules. Piezo1, a mechanosensitive nonselective cation channel, is broadly expressed across diverse cell types and tissues, enabling the direct transduction of mechanical forces into cellular electrochemical signals. In recent years, the involvement of Piezo1 in the pathogenesis of fibrotic diseases has attracted considerable research interest. Studies have demonstrated that Piezo1 contributes to the fibrotic process across multiple organs by mediating various signalling pathways [17, 18, 19], indicating that Piezo1 is a promising therapeutic target for the treatment of fibrotic diseases.

Therefore, the aim of this study was to investigate the role and underlying mechanism of Piezo1 in MV‐induced diaphragm fibrosis. This study reveals, for the first time, in a rat model of MV, that periodic mechanical stress mediates diaphragmatic fibrosis by increasing the expression of the mechanically sensitive ion channel Piezo1 in the diaphragm. The Akt/Nr4a1 signalling pathway may play a critical role in this pathological process. These findings not only highlight the critical role of Piezo1 in diaphragmatic dysfunction induced by prolonged MV but also offer a novel theoretical framework for understanding the molecular mechanisms underlying diaphragmatic injury mediated by mechanical force transduction.

## 2 Methods

### 2.1 Animals and Experimental Design

Male Sprague–Dawley (SD) rats aged 8–12 weeks (250–300 g) were purchased from the Animal Experiment Center of Southwest Medical University. Animals were maintained on a 12:12 h light:dark cycle and were provided with food and water ad libitum throughout the experiment. All of our experimental procedures were performed in accordance with the Institutional Guidelines for the Use and Care of Laboratory Animals. Animal experiments were approved by the Ethics Committee of the Animal Experiment Center of Southwest Medical University (No. swum 20240072).

#### 2.1.1 Experiment 1

This study aims to investigate the effects of different durations of MV on the structure and function of the diaphragm, as well as the expression of Piezo1 in the diaphragm of rats. The animals were randomly allocated into three groups using a random number table: (1) the control group (*n* = 6), which received no MV; (2) the MV6 group (*n* = 6), which was subjected to 6 h of MV; and (3) the MV12 group (*n* = 6), which was subjected to 12 h of MV.

#### 2.1.2 Experiment 2

To investigate whether GsMTx4 (a Piezo1‐nonspecific inhibitor) can alleviate diaphragm fibrosis induced by prolonged MV, SD rats were randomly assigned to one of three experimental groups: (1) the control group (*n* = 5), which received no MV; (2) the MV12 group (*n* = 5), which was subjected to 12 h of MV; and (3) the MV12 + GsMTx4 group (*n* = 5), which was subjected to 12 h of MV and treated with GsMTx4.

#### 2.1.3 Experiment 3

To investigate whether adeno‐associated virus (AAV)‐Piezo1 knockdown attenuates the progression of diaphragm fibrosis induced by MV, SD rats were randomly assigned to four experimental groups: (1) the control + shCtrl group (*n* = 5), which was transfected with an empty plasmid without the AAV target gene; (2) the Control + shPiezo1 group (*n* = 5), which was transfected with the AAV target gene plasmid Piezo1; (3) the MV12 + shCtrl group (*n* = 5), which was subjected to 12 h of MV and transfected with an empty plasmid without the AAV target gene; and (4) the MV12 + shPiezo1 group (*n* = 5), which was subjected to 12 h of MV and transfected with the AAV target gene plasmid Piezo1.

#### 2.1.4 Experiment 4

To investigate whether cytosporone‐B (Csn‐B, a specific agonist of Nr4a1) treatment can attenuate diaphragm fibrosis induced by prolonged MV, SD rats were randomly assigned to one of three experimental groups: (1) the control group (*n* = 5), which received no MV; (2) the MV12 group (*n* = 5), which was subjected to 12 h of MV; and (3) the MV12 + Csn‐B group (*n* = 5), which was subjected to 12 h of MV and treated with Csn‐B. The control and MV12 groups in this experiment are the control and MV12 groups in Experiment 2.

### 2.2 MV Treatment

Rats were administered an intraperitoneal injection of sodium pentobarbital (60 mg/kg), followed by the insertion of a 24‐G indwelling needle via tail vein puncture. Continuous infusion was maintained at a rate of 10 mg/kg/h of sodium pentobarbital and 2 mL/kg/h of saline. The animals were placed on an operating table in the supine position and secured, with 1% lidocaine applied to the neck incision site to mitigate pain. A midline neck incision was made for tracheostomy, and a small animal ventilator (Rivard, China) operating in volume‐controlled mode was connected. The ventilator parameters were set as follows: tidal volume (Vt) = 10 mL/kg, peak inspiratory pressure (PIP) = 25 cmH_2_O, inspiratory to expiratory ratio (I:E) = 1:2, fraction of inspired oxygen (FiO_2_) = 21%, positive end‐expiratory pressure (PEEP) = 0 and respiratory rate (RR) = 70–80 bpm, which could be adjusted appropriately for the rats. Arterial blood samples were obtained at the end of the experiment to monitor arterial blood gas. During prolonged MV, continuous care for the rats included maintaining a body temperature of 37°C, lubricating the eyes, expressing the bladder, clearing airway mucus and rotating the rats to prevent blood stasis.

### 2.3 Survival Surgery for AAV9‐Piezo1 Administration

AAV was procured from Hanbio (HH20240709CDNWH‐AAV01, China). Four weeks prior to MV, rats were administered either a plasmid containing the gene of interest (AAV‐Piezo1) or an empty transgenic carrier plasmid (AAV‐shRNA) directly into the diaphragm. This time point was selected based on the results of literature reports [20, 21] and preliminary experiments, indicating that 4 weeks after transfection was sufficient to result in a stable knockdown of the target gene (Table S1). The complete details of the diaphragm gene transfection protocol are provided by Smuder et al. [22]. Rats were anaesthetized with 60 mg/kg sodium pentobarbital. Under sterile conditions, a 4‐cm midline incision (xiphoid to suprapubic) was made. The diaphragm was exposed by elevating the xiphoid and retracting the liver. Eight injections (40 μL each, ~2 × 10^11^ vg total AAV) were evenly distributed across the diaphragm, with tissue blebs confirming successful delivery.

### 2.4 Measurement of Diaphragm Frequency‐Contraction Curve and Fatigue Index

The ribbed muscle strips approximately 3 mm wide and 1 cm long were dissected from the costal diaphragm, with one end attached to the ribs and the other to the central tendon. The strips were immersed in a cyclically heated Krebs–Hensleit solution, which was equilibrated with a gas mixture of 95% O_2_ and 5% CO_2_ and maintained at 37°C with a pH of 7.4. One end of the muscle strip was secured to a force transducer using a suture, whereas the opposite end was fixed in place. The stimulation voltage, duration and interstimulation interval were set at 15 V, 2 ms and 3 min, respectively. The optimal length was determined by systematically adjusting myofilament length to achieve peak active force. The muscle was then continuously stimulated with a 15‐V stimulation voltage at frequencies of 10, 20, 40, 60, 80, 100 and 120 Hz for 1 s, with a 1‐min interval between each stimulation, to determine the frequency–contraction curve relationship. The lengths and weights of the muscle strips were measured. Muscle density was calculated as 1.06 g/cm^3^, and the CSA of the muscle strip was calculated as CSA = weight/density/length. The contractile force of the myofilament was normalized by the CSA, expressed as force per unit area (g/cm^2^).

After the optimal contraction length was achieved in a second costal muscle strip, the muscle was continuously stimulated for 120 s at 50 Hz using a pulsed square wave (intensity 15 V, pulse width 0.5 ms and delay 20 ms). The fatigue index is defined as the ratio of the initial force magnitude to the force magnitude generated after 120 s.

### 2.5 Real‐Time Quantitative Polymerase Chain Reaction (RT‐qPCR)

TRIzol reagent (Tiangen, China) was utilized to extract total RNA from diaphragm tissue. Complementary DNAs (cDNAs) were synthesized using a quantitative polymerase chain reaction (qPCR) kit (Vazyme, China). Experiments were conducted in accordance with the manufacturer's protocol. The target genes included Piezo1, Piezo2, Trpv1, Trpv4 and Trpc1. GAPDH served as the reference housekeeping gene, and the relative expression levels of the target genes were determined using the 2^−ΔΔCt^ method. The primer sequences are provided in Table S2.

### 2.6 Western Blot Analysis

The diaphragm samples were lysed with RIPA buffer for 30 min, and the proteins were extracted. The proteins were separated by electrophoresis on a sodium dodecyl sulphate‐polyacrylamide gel electrophoresis (SDS‐PAGE). The proteins were transferred to nitrocellulose membranes under a steady current of 220 mA. The membrane was blocked with milk at room temperature for 90 min and incubated with primary antibodies against piezo1, fibronectin (FN), Collagen Type I Alpha 1 (Col1a1), Collagen III (Col3), α‐smooth muscle actin (α‐SMA), Nr4a1, Akt 1, Phospho‐Akt (P‐Akt) and GAPDH (Table S3) overnight at 4°C, followed by incubation with secondary antibodies for 60 min.

### 2.7 RNA‐seq Analysis

For RNA‐seq, three biological replicates per group (shPiezo1 + MV12 and shCtrl + MV12) were analysed. Total RNA was extracted, quality‐checked (Agilent 5300 Bioanalyzer) and quantified (NanoDrop ND‐2000). RNA purification, library preparation and Illumina sequencing were conducted by Majorbio Bio‐pharm (Shanghai, China). Subsequently, differential expression analysis and functional enrichment analysis, including Gene Ontology (GO) and Kyoto Encyclopedia of Genes and Genomes (KEGG), were performed.

### 2.8 Staining

The diaphragm samples were fixed using an environmentally friendly GD muscle fixing solution (G1111, Servicebio) and subsequently embedded in paraffin.

#### 2.8.1 Masson Staining

Sections were stained using Masson's Trichrome (DC0032, Leagene) as follows: Weigert's iron haematoxylin for 5 min, acidic ethanol differentiation for 5 s, Masson Blue solution for 5 min, Ponceau Staining solution for 10 min, phosphomolybdic acid solution for 30 s and Aniline Blue solution for 2 min, with intermittent distilled/weak acid washes. Sections were then dehydrated, cleared and mounted.

#### 2.8.2 Sirius Red Staining

Sections were stained using the Sirius Red Stain Kit (G1078, Servicebio) as follows: incubated in Solution A (65°C, 30 min), rinsed until tissue yellowing faded, treated with Solution B (2 min) and Solution C (30 min) and then briefly washed, dehydrated, cleared and mounted.

#### 2.8.3 Immunohistochemistry

Following antigen retrieval, the procedure was conducted according to the protocol specified in the M&R HRP/DAB Detection IHC Kit (HC301 Vazyme). Briefly, after blocking endogenous peroxidase with hydrogen peroxide blocking reagent, the sections were incubated with the primary antibody overnight at 4°C. Following PBS wash steps, the sections were incubated with HRP polymer at room temperature for 20 min. Subsequently, the sections underwent staining with 3, 3′‐diaminobenzidine (DAB, a peroxidase substrate producing brown precipitate) and haematoxylin counterstaining.

#### 2.8.4 Immunofluorescence

The sections were permeabilized with 0.2% Triton X‐100 and subsequently blocked with 5% BSA at room temperature for 1 h. The sections were subsequently incubated with primary antibodies against α‐SMA (Table S3) overnight at 4°C. After washing, the sections were incubated with anti‐mouse Alexa Fluor 488 (CST) secondary antibodies (1:500) at room temperature.

### 2.9 Statistical Analysis

Image J was utilized to process and analyse the images, and GraphPad Prism 10 software was employed for statistical analysis. Data are presented as the mean ± standard error. Independent sample *t* test was used to compare the mRNA expression levels of Piezo2, Trpv1, Trpv4 and Trpc1 between CON and MV12 groups. A one‐way analysis of variance (ANOVA) was conducted to compare three or more groups, followed by post hoc testing using Tukey's test. *p* < 0.05 was considered statistically significant.

## 3 Results

### 3.1 Prolonged MV Leads to Fibrosis and Dysfunction of the Diaphragm in Rats

**FIGURE 1:**
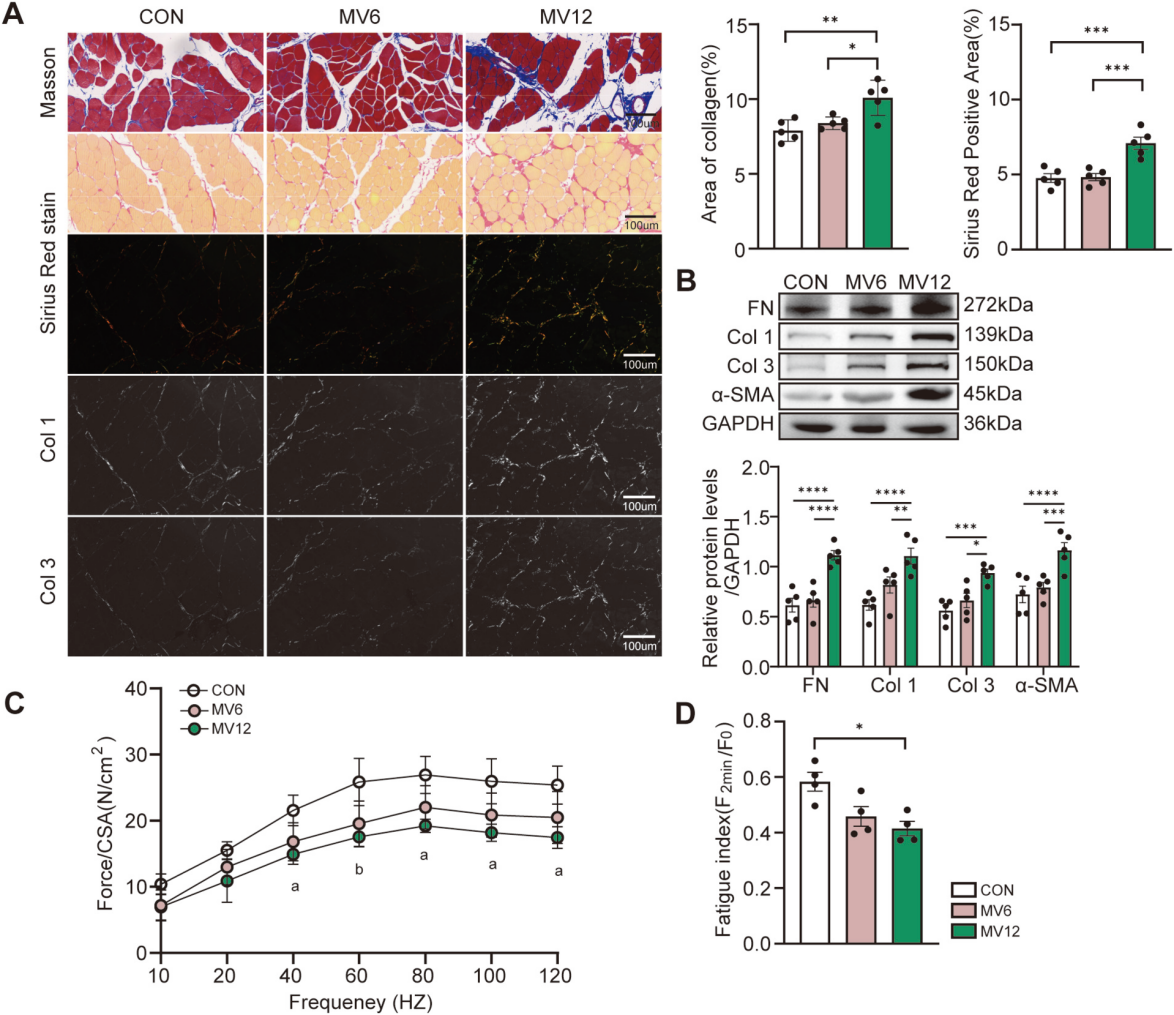
Prolonged MV leads to fibrosis and dysfunction of the diaphragm in rats. (A) Representative images (400X magnification) of Masson staining and Sirius Red staining (bright and dark fields). Row 1: Masson staining (muscle: red; collagen: blue). Row 2: Sirius Red staining under bright field (muscle: yellow; collagen: red). Row 3: Sirius Red staining under dark field (collagen: yellow‐green). Rows 4 and 5: decomposed images from Row 3, representing Collagens I and III, respectively. Scale bar = 100 μm. Positive staining colours (blue for Masson and red for Sirius Red) were extracted and analysed quantitatively. Five fields of view were randomly selected per slice, and the results were averaged. (B) Expression levels of GAPDH, FN, Col 1, Col 3 and α‐SMA were assessed via western blotting and quantitative protein analysis. (C) The force‐frequency curve of the diaphragm muscle is shown; ‘a’ denotes *p* < 0.01 for the comparison between MV12 and CON, whereas ‘b’ indicates *p* < 0.05 for MV6 versus CON and *p* < 0.01 for MV12 versus CON. (D) The diaphragm fatigue index was measured for each group. Data are presented as mean ± SE. Each group included no fewer than three biological replicates. One‐way ANOVA was applied for all analyses, with statistical significance indicated as follows: *p < 0.05, **p < 0.01 and ***p < 0.005. α‐SMA, alpha‐smooth muscle actin; ANOVA, analysis of variance; Col 1, Collagen Type I; Col 3, Collagen Type III; FN, fibronectin; MV, mechanical ventilation; SE, standard error.

Table S4 presents the body weight and arterial blood gas parameters of the rats, indicating no statistically significant differences among the groups (all *p* > 0.05). Masson's trichrome and Sirius Red staining demonstrated a significantly increased collagen area in the MV12 group compared to both the control and MV6 groups (all *p* < 0.05, Figure 1A). Western blot analysis further confirmed that 12 h of MV significantly upregulated the expression of fibrosis‐associated markers in the diaphragm, including ECM components FN, Col 1, Col 3 and the myofibroblast activation marker α‐SMA (all *p* < 0.05, Figure 1B). These findings indicate that MV‐induced diaphragm fibrosis progresses in a time‐dependent manner. Meanwhile, compared with the control group, the cross‐sectional area (CSA) of diaphragm muscle fibres in the MV12 group was markedly reduced (*p* = 0.0009, Figure S3A). In the evaluation of isolated diaphragm function, the frequency‐contraction curve of the MV12 group was significantly shifted downward, with the contraction force markedly reduced compared with the control group during high‐frequency stimulation (40–120 Hz) (all *p* < 0.05, Figure 1C). Consistent with these findings, the fatigue index of the MV12 group was lower than that of the control group, indicating impaired fatigue resistance in the diaphragm (*p* = 0.012, Figures 1D and S1B). These data indicate that 12 h of MV leads to the development of fibrosis, atrophy and dysfunction in the rat diaphragm.

### 3.2 Upregulation of Piezo1 Expressions in Diaphragm Fibrosis Induced by MV

**FIGURE 2:**
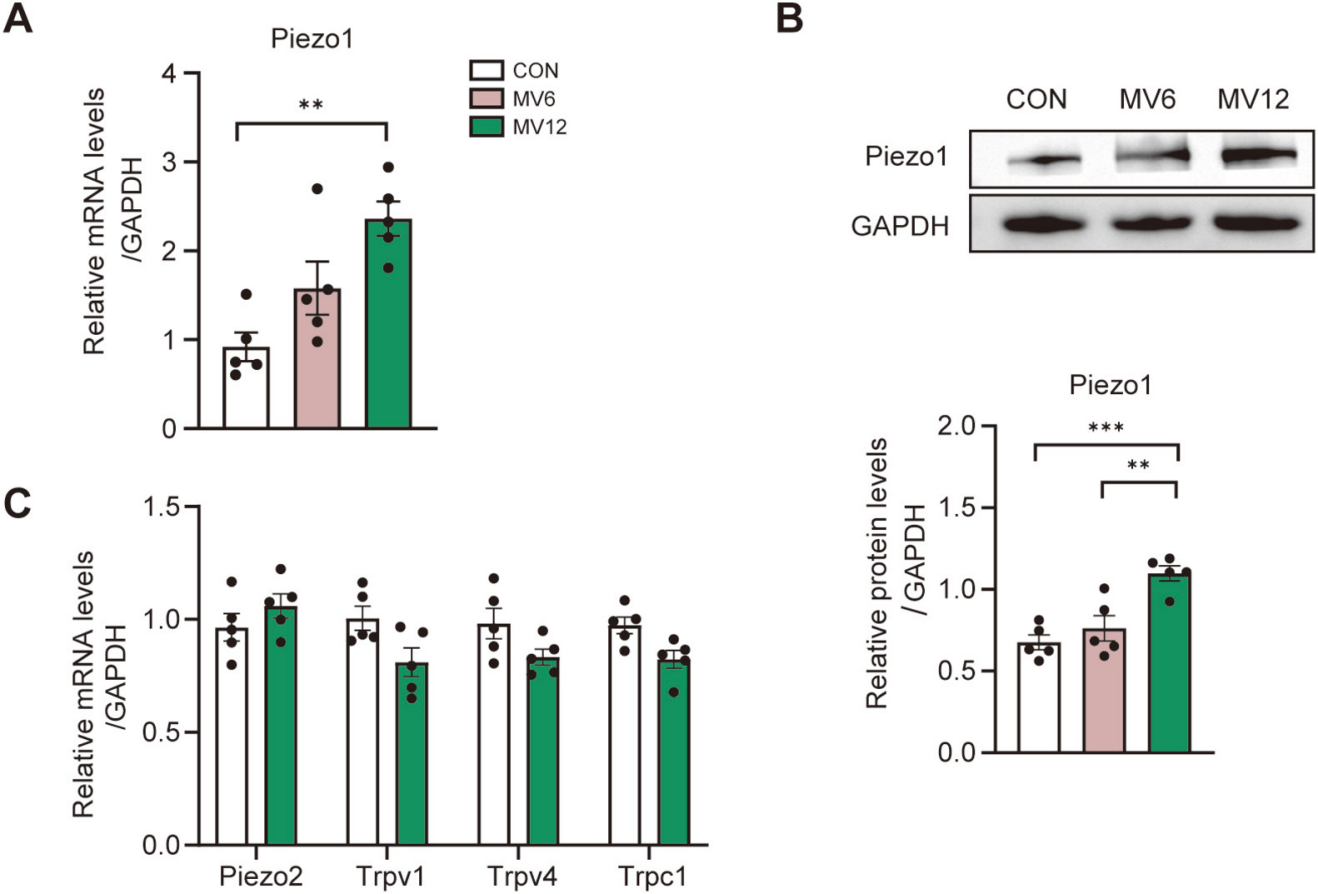
Upregulation of Piezo1 expressions in diaphragm fibrosis induced by MV. (A) mRNA levels of Piezo1 in the diaphragm across groups. (B) Western blot analysis of Piezo1 protein levels in the diaphragm of each group. (C) mRNA levels of Piezo2, Trpv1, Trpv4 and Trpc1 in the diaphragm of the CON and MV12 groups. Data are presented as mean ± SE. Independent sample *t* test was used to compare the two groups, and one‐way ANOVA was conducted to compare three, with statistical significance indicated as follows: **p < 0.01 and ***p < 0.005.

To explore the potential association between Piezo1 expression and diaphragm fibrosis, Piezo1 levels were assessed in diaphragms subjected to different durations of MV. Piezo1 expression was significantly upregulated after 12 h of MV, with mRNA levels increasing 2.6‐fold (*p* = 0.0018, Figure 2A) and protein levels rising 1.7‐fold (*p* = 0.0007, Figure 2B) compared with the control group. In contrast, the mRNA levels of Trpv1, Trpv4 and Trpc1 were unchanged after 12 h of MV (all *p* > 0.05, Figure 2C). Moreover, the mRNA level of Piezo2, another member of the Piezo family, was assessed, revealing no significant difference between the MV12 group and the control group (*p* = 0.6162, Figure 2C). These findings suggest that prolonged MV may promote diaphragm fibrosis and dysfunction, potentially through Piezo1‐specific upregulation.

### 3.3 GsMTx4 Alleviates MV‐Induced Diaphragm Fibrosis

**FIGURE 3:**
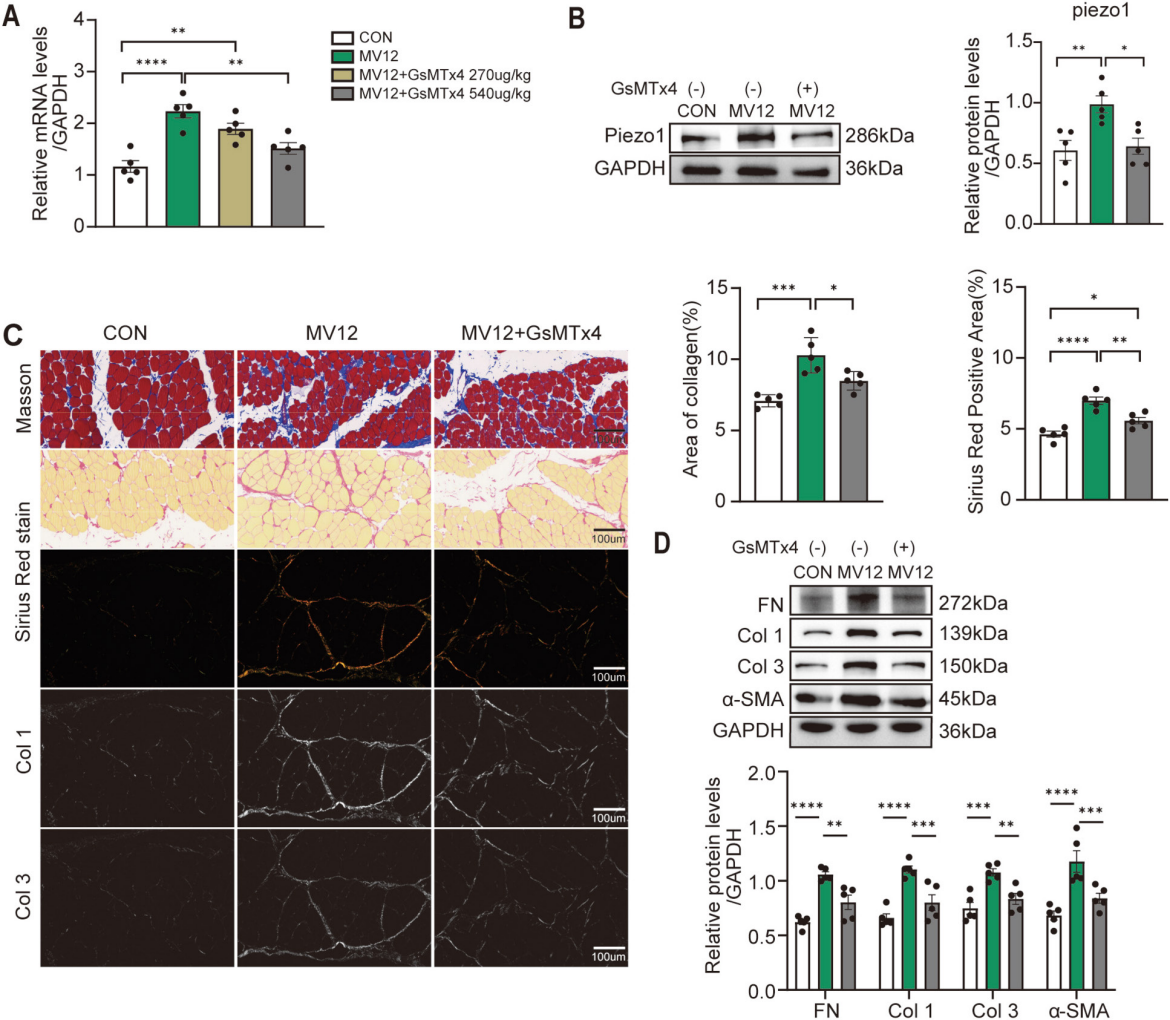
GsMTx4 alleviates MV‐induced diaphragm fibrosis. (A) mRNA levels of Piezo1 in the diaphragm of each group. (B) Western blot analysis of Piezo1 protein levels in the diaphragm of each group. (C) Representative images (400X magnification) of Masson and Sirius Red staining. Scale bar = 100 μm. Five fields of view were randomly selected per slice, and the results were averaged. (D) Expression levels of GAPDH, FN, Col 1, Col 3 and α‐SMA were assessed via western blotting and quantitative protein analysis. Data are presented as mean ± SE. Each group included no fewer than three biological replicates. One‐way ANOVA was applied for all analyses, with statistical significance indicated as follows: *p < 0.05, **p < 0.01, ***p < 0.005 and ****p < 0.001.

We subsequently investigated whether the inhibition of Piezo1 improved MV‐induced diaphragm fibrosis. A single intraperitoneal injection of 270 or 540 μg/kg GsMTx4, a nonspecific Piezo1 inhibitor, was administered 30 min prior to MV. GsMTx4 (540 μg/kg) significantly reduced the mRNA level of Piezo1 in the diaphragm of MV12 rats (*p* = 0.0023, Figure 3A); thus, this dosage was selected. GsMTx4 also reduced the protein expression level of Piezo1 in the diaphragm of MV12 rats (*p* = 0.0147, Figure 3B). The ability of GsMTx4 to improve fibrosis was confirmed by Masson's trichrome staining and Sirius Red staining. Compared to the control group, the MV12 group exhibited more extensive fibrotic lesions, characterized by intensified collagen staining, whereas treatment with GsMTx4 significantly reduced the fibrotic area in the MV12 diaphragm (all *p* < 0.05, Figure 3C). GsMTx4 partially reversed MV12‐induced upregulation of fibrotic markers (all *p* < 0.05, Figure 3D) and mitigated diaphragm atrophy (*p* = 0.0437, Figure S3B). Collectively, these results indicate that GsMTx4‐mediated inhibition of Piezo1 partially mitigates diaphragm fibrosis and atrophy induced by prolonged MV.

### 3.4 AAV‐Mediated Piezo1 Knockdown Attenuates the Progression of Diaphragm Fibrosis Induced by MV

**FIGURE 4:**
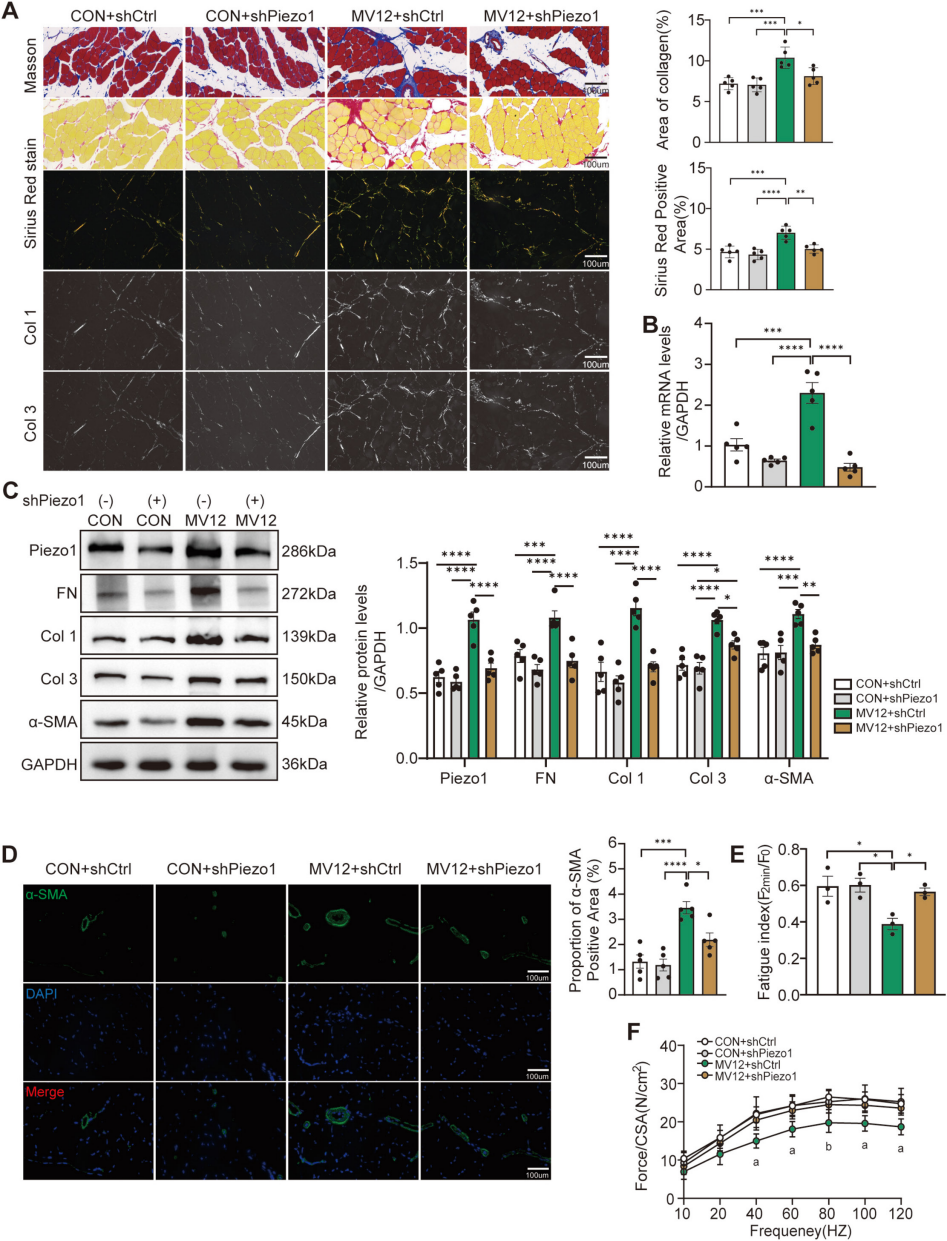
AAV‐mediated Piezo1 knockdown attenuates MV‐induced diaphragm fibrosis. (A) Representative images (400X magnification) of Masson and Sirius Red staining. Scale bar = 100 μm. Five fields of view were randomly selected for each slice, and the results were averaged. (B) Western blot analysis of Piezo1, FN, Col 1, Col 3 and α‐SMA protein levels in the diaphragm across groups. Expression levels were normalized to GAPDH. (C) Immunofluorescence images of α‐SMA (green) in diaphragm of each group and quantitative analysis of positive signals. Scale bar = 100 μm. (D) Fatigue index of the diaphragm was assessed for all groups. (E) The diaphragm force‐frequency relationship is depicted; ‘a’ indicates that MV12 + shCtrl group differs significantly from CON + shCtrl, CON + shPiezo1 and MV12 + shPieoz1 groups (*p* < 0.05). ‘b’ denotes *p* < 0.05 for comparisons of both CON + shPiezo1 and MV + shPiezo1 to MV + shCtrl, and *p* < 0.01 for CON + shCtrl versus MV + shCtrl. Data are expressed as mean ± SE. Each group included a minimum of three biological replicates. Statistical analysis was performed using one‐way ANOVA, with significance thresholds defined as *p < 0.05, **p < 0.01, ***p < 0.005 and ****p < 0.001.

As GsMTx4 is not a specific inhibitor of Piezo1, AAV‐mediated RNA interference (shPiezo1) was employed to further elucidate the pivotal role of Piezo1 in MV‐induced diaphragm fibrosis. Compared to the MV12 + shCtrl group, the MV12 + shPiezo1 group exhibited a significant reduction in diaphragm fibrosis, as indicated by about a 25% decrease in collagen deposition area (all *p* < 0.05, Figure 4A) and a marked decline in the expression of fibrosis‐associated marker proteins (all *p* < 0.05, Figure 4C). Consistent with these findings, AAV‐shPiezo1 treatment significantly inhibited the upregulation of Piezo1 mRNA and protein expression following 12 h of MV (all *p* < 0.05, Figure 4B,C). Immunofluorescence analysis confirmed that AAV‐shPiezo1 treatment significantly reduced MV‐induced α‐SMA expression (*p* = 0.0125, Figure 4D). Furthermore, Piezo1 knockdown alleviated MV‐induced diaphragm atrophy (*p* = 0.002, Figure S3C) and improved muscle function, enhancing both fatigue resistance (*p* = 0.0465, Figures 4E and S1C) and contractility (all *p* < 0.05, Figure 4F). These results indicate that AAV‐mediated Piezo1 knockdown effectively mitigates diaphragm fibrosis, atrophy and functional impairment induced by prolonged MV.

### 3.5 Piezo1 Mediates MV‐Induced Diaphragm Fibrosis via the Akt/Nr4a1 Axis

**FIGURE 5:**
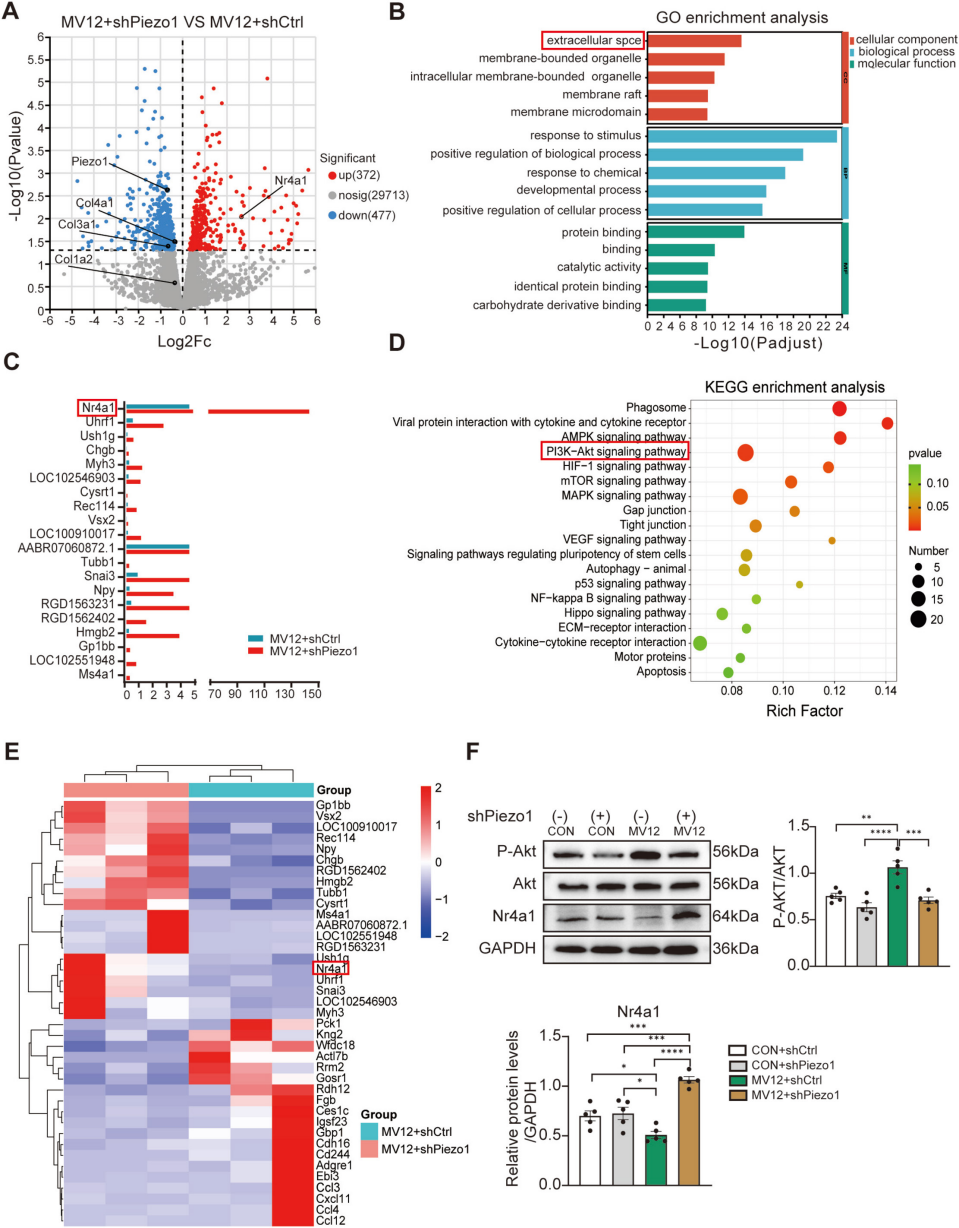
Piezo1 may mediate MV‐induced diaphragm fibrosis via the Akt/Nr4a1 axis. (A) Volcano plot showing DEGs between the MV12 + shPiezo1 and MV12 + shCtrl groups. (B) GO enrichment analysis of DEGs between the MV12 + shPiezo1 and MV12 + shCtrl groups. (C) Top 20 downregulated genes in the MV12 + shPiezo1 group, ranked by log2FC. (D) KEGG pathway enrichment analysis of DEGs between the MV12 + shPiezo1 and MV12 + shCtrl groups. (E) Heatmap illustrating hierarchical clustering of DEGs between the MV12 + shPiezo1 and MV12 + shCtrl groups. (F) Western blot analysis of GAPDH, P‐Akt/Akt and Nr4a1 protein levels in the diaphragm of each group. Data are presented as mean ± SE. Each group included three independent biological replicates. One‐way ANOVA was used for statistical analysis. Significance is denoted as follows: *p < 0.05, **p < 0.01 and ****p < 0.001. Abbreviations: DEGs, differentially expressed genes; GO, Gene Ontology; KEGG, Kyoto Encyclopedia of Genes and Genomes.

Subsequently, to further elucidate the molecular mechanisms underlying Piezo1‐mediated diaphragm fibrosis, whole transcriptome sequencing was performed on diaphragm tissues from the MV12 + shPiezo1 and MV12 + shCtrl groups. Among 30 562 genes analysed, 849 showed differential expression (372 upregulated and 477 downregulated) following Piezo1 knockdown (Figure 5A). Consistent with our hypotheses, Piezo1 knockdown markedly decreased the expression levels of ECM‐associated genes (Figure 5A). Gene Ontology (GO) enrichment analysis revealed significant enrichment of the term ‘extracellular space’ within the cellular component (CC) category, underscoring the critical role of ECM remodelling in fibrosis (Figure 5B). Notably, Nuclear Receptor Subfamily 4 Group A Member 1 (Nr4a1) was ranked 20th among the upregulated DEGs (Figure 5C). Beyond Nr4a1, Figure 5E highlights several transcripts—Gp1bb (platelet glycoprotein Ib beta chain), Vsx2 (visual system homeobox 2), Npy (neuropeptide Y) and Tubb1 (tubulin beta‐1 chain)—that show clear group separation. Although our study was not designed to assess each gene's individual role, Piezo1 silencing may partly alleviate VIDD by affecting these pathways. Previous studies have demonstrated that Nr4a1 exerts antifibrotic effects by inhibiting the TGF‐β/Smad signalling pathway [23]. Further Kyoto Encyclopedia of Genes and Genomes (KEGG) pathway enrichment analysis revealed significant coenrichment of the ‘PI3K‐AKT signalling pathway’ and ECM‐related genes (Figure 5D). Previous studies have demonstrated that mechanical stimulation can induce Akt phosphorylation through the activation of Piezo1 [24] and that the Akt signalling pathway suppresses both the expression and transcriptional function of Nr4a1 [25, 26]. Based on these findings, we hypothesized that the MV‐induced upregulation of Piezo1 drives diaphragm fibrosis via the Akt/Nr4a1 axis. Western blot analysis revealed that the ratio of phosphorylated Akt (P‐Akt) to total Akt was significantly elevated in the MV12 + shCtrl group compared to the control group (*p* = 0.0017, Figure 5F), whereas Nr4a1 protein expression was notably reduced (*p* = 0.0475, Figure 5F). Conversely, AAV‐shPiezo1 treatment markedly inhibited Akt phosphorylation and resulted in upregulated Nr4a1 expression (all *p* < 0.05, Figure 5F). These findings suggest that Nr4a1 may serve as a critical downstream target of Piezo1.

### 3.6 Csn‐B Ameliorates Diaphragm Fibrosis Induced by MV

**FIGURE 6:**
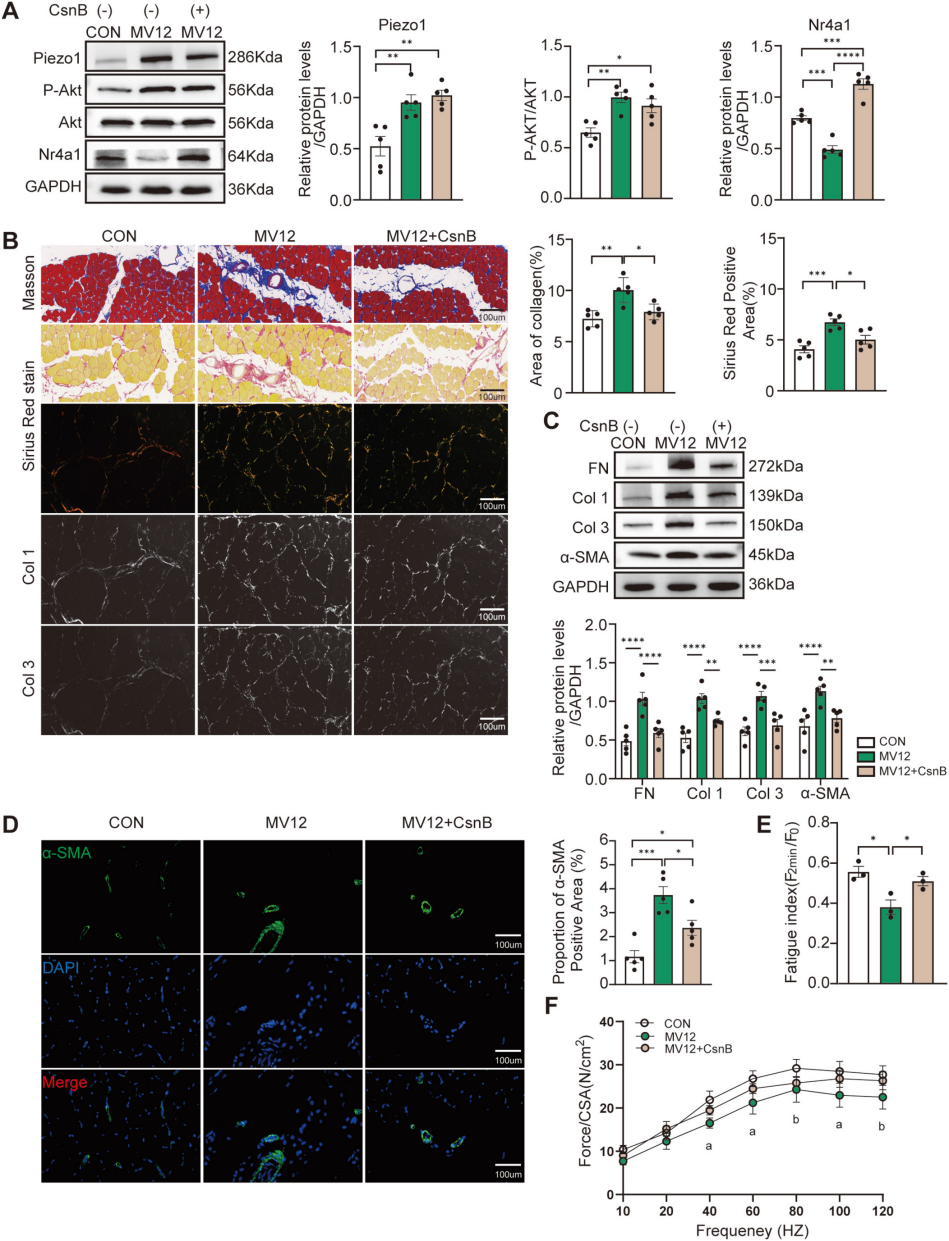
Csn‐B ameliorates diaphragm fibrosis induced by MV. (A) Western blot analysis of GAPDH, Piezo1, P‐Akt/Akt and Nr4a1 protein levels in the diaphragm of each group. (B) Representative images (400X magnification) of Masson and Sirius Red staining. Scale bar = 100 μm. Five randomly selected fields per slice were averaged for analysis. (C) Western blot analysis of FN, Col 1, Col 3 and α‐SMA protein levels in the diaphragm of each group. The results were normalized to the expression of GAPDH. (D) Immunofluorescence images of α‐SMA (green) in the diaphragm of each group and quantitative analysis of positive signals. Scale bar = 100 μm (E) Diaphragm fatigue index for each group. (F) Force‐frequency curve of diaphragm muscle; ‘a’ indicates *p* < 0.01 for CON versus MV12, and ‘b’ indicates *p* < 0.05 for CON versus MV12. Data are expressed as mean ± SE. Each group included three independent biological replicates. One‐way ANOVA was used for all analyses. Significance is denoted as follows: *p < 0.05, **p < 0.01, ***p < 0.005 and ****p < 0.001.

Given that Piezo1 may mediate MV‐induced diaphragm fibrosis through the suppression of Nr4a1 expression, the therapeutic potential of targeting this pathway was evaluated using Csn‐B. Csn‐B was administered intraperitoneally 30 min prior to the initiation of MV, and its antifibrotic effects were systematically assessed. Intervention with Csn‐B significantly elevated Nr4a1 expression (*p* < 0.0001, Figure 6A) without affecting Piezo1 (*p* = 0.794, Figure 6A) or P‐Akt/Akt levels (*p* = 0.552, Figure 6A) compared to the MV12 group. Compared with the MV12 group, the area of collagen deposition in the MV12 + CsnB group was significantly decreased (all *p* < 0.05, Figure 6B), and the relative protein abundance of FN, Col 1, Col 3 and α‐SMA was notably decreased (all *p* < 0.05, Figure 6C). Consistent with these findings, immunofluorescence and histochemical analyses further demonstrated that Csn‐B effectively reduced the expression level of α‐SMA induced by MV (all *p* < 0.05, Figures 6D and S2A,B). Compared with the MV12 group, the MV12 + Csn‐B group showed significantly enhanced fatigue resistance (*p* = 0.046, Figures 6E and S1D). However, no significant differences were observed in the frequency‐contraction curves between the MV12 + Csn‐B group and either the CON or MV12 groups (all *p* > 0.05, Figure 6F). These findings suggest that Csn‐B may improve diaphragm function despite a nonsignificant gain in fibre size (*p* = 0.108, Figure S3D). These findings suggest that Csn‐B treatment partially alleviates MV‐induced fibrosis and dysfunction in the diaphragm.

## 4 Discussion

**FIGURE 7:**
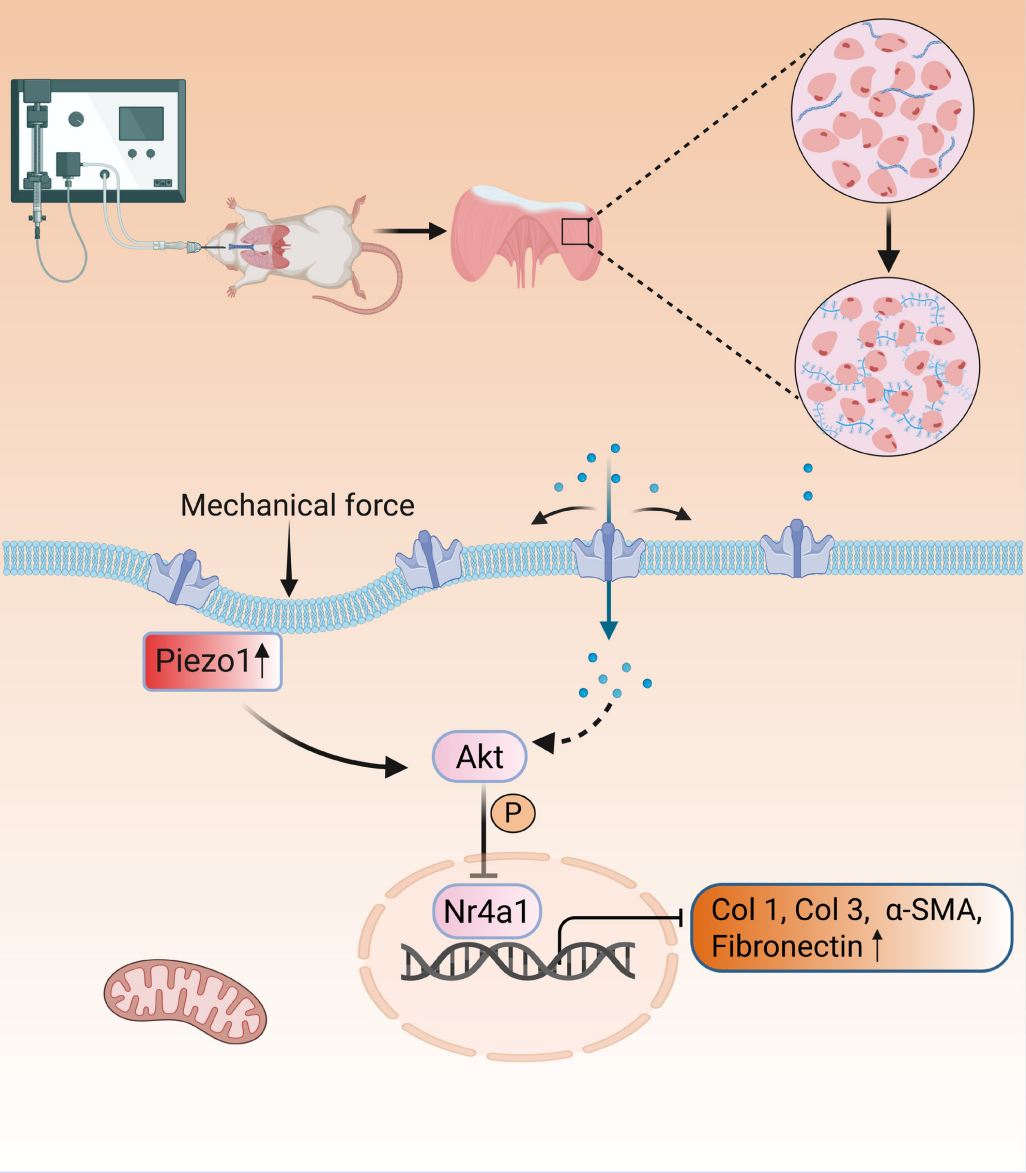
Schematic working model of the study. Prolonged mechanical ventilation may mediate diaphragm fibrosis and dysfunction through upregulation of Piezo1 expression in the diaphragm, with the P‐Akt/Nr4a1 signalling pathway potentially playing a key regulatory role in this pathological process.

This study uncovers a novel molecular mechanism underlying diaphragm fibrosis induced by prolonged MV and identifies the mechanosensitive ion channel Piezo1 as a critical regulatory factor in this pathological process (Figure 7).

VIDD has traditionally been attributed to disuse atrophy of the diaphragm. Interestingly, clinical studies have reported that the thickness of the diaphragm in MV patients may decrease, remain unchanged or even increase [27, 28], suggesting that the pathological mechanism of VIDD is more complex. Research has revealed a significant accumulation of ECM in the diaphragms of critically ill patients undergoing MV, leading to rapid fibrosis [7, 8]. Although the ECM is an essential component of the skeletal muscle microenvironment, accounting for 5%–10% of skeletal muscle mass and playing a crucial role in force transmission, structural maintenance and muscle fibre repair [29], its abnormal accumulation, particularly of collagen, can increase skeletal muscle stiffness and fragility, thereby limiting muscle elasticity and contraction efficiency [30]. These observations indicate that fibrosis not only reflects structural alterations in the diaphragm but also constitutes a critical contributor to the functional impairment associated with VIDD. However, the detrimental impact of diaphragm fibrosis on diaphragmatic function has been largely underestimated in previous studies.

Animal studies have identified asynchronous MV and the use of elevated tidal volumes or PEEP as key contributors to the development of diaphragm fibrosis and impaired function [11, 31, 32]. In our study, MV with a tidal volume of 10 mL/kg for 12 h resulted in the development of diaphragm fibrosis, which was accompanied by decreased contractility and reduced fatigue resistance. Consistent with fibrosis, Piezo1 mRNA and protein levels were significantly increased with prolonged MV, confirming previous findings that mechanical stress can stimulate Piezo1 expression in different cell types [33, 34, 35]. Notably, the mRNA levels of other mechanosensitive ion channels, such as Trpv1, Trpv4, Trpc1 and Piezo2, did not significantly change with prolonged MV. Interestingly, this study found that Piezo1 expression was significantly higher in the MV12 group than in the MV6 group, but the fatigue index did not differ between the two groups. This suggests that Piezo1 upregulation may precede detectable diaphragm dysfunction, which likely results from multiple factors. It is important to note that GsMTx4 acts as a blocker of cationic mechanosensitive channels by inhibiting the activation of ion channels. However, in our study, GsMTx4 suppressed the upregulation of Piezo1 induced by prolonged MV, although the precise molecular mechanism underlying this effect remains to be elucidated. This finding is consistent with the results reported by Zhao et al. [36]. In a renal fibrosis model, GsMTx4 effectively attenuated the upregulation of Piezo1 in the kidney. These results suggest that the upregulation of Piezo1 expression may play a crucial role in the development of diaphragm fibrosis. This effect may be attributed to Piezo1's function as a nonselective cation channel, capable of directly sensing mechanical forces, leading to the influx of extracellular Ca^2+^ ions and the subsequent activation of intracellular signalling cascades [37, 38].

Previous studies have established that mechanical stress activates Akt signalling through Piezo1, a pathway implicated in cardiovascular protection, tumour progression and fibrotic disease [39, 40]. Akt phosphorylation markedly reduces Nr4a1 expression and transcriptional activity through multiple pathways [25, 26]. Our interest lies in elucidating the relationships among Piezo1, Nr4a1 and fibrosis. Nr4a1 has significant tissue‐specific effects during fibrosis. On the one hand, the activation of Nr4a1 alleviated renal interstitial fibrosis resulting from ureteral obstruction [41]. On the other hand, Nr4a1 has also been shown to activate the p38 MAPK signalling pathway to promote renal fibrosis [42] or to modulate the phenotypes of cardiac fibroblasts and macrophages, thereby facilitating angiotensin II‐induced cardiac fibrosis [43]. In the present study, Csn‐B, which specifically binds to the ligand‐binding domain of Nr4a1 and stimulates Nr4a1‐dependent transcription of target genes, including Nr4a1 itself [44, 45, 46], significantly ameliorated diaphragm fibrosis and dysfunction induced by prolonged MV. This therapeutic effect is likely attributable to the combined actions of TGF‐β signalling inhibition [23], modulation of macrophage phenotype [47], and promotion of myoblast differentiation and fusion [48], which together enhance muscle contractility and fatigue resistance. AAV‐mediated Piezo1 knockdown elevated Nr4a1 expression, indicating that Piezo1 might indirectly modulate Nr4a1 through Akt phosphorylation. However, CsnB‐induced upregulation of Nr4a1 did not alter Piezo1 expression, implying that Nr4a1 can be regulated independently of Piezo1. Further studies are needed to fully elucidate the molecular interactions between Piezo1, Akt and Nr4a1 in MV‐induced diaphragmatic dysfunction.

Interestingly, the findings indicated that MV‐induced diaphragm fibrosis exhibits distinct pathological characteristics, primarily marked by the deposition of ECM in the interstitial and perivascular areas. Immunofluorescence and histochemical analyses showed a circular pattern of α‐SMA distribution. We speculate that the possible underlying mechanisms are as follows: During diaphragm fibrosis, myofibroblasts may be selectively activated and recruited to perivascular regions, where they form ring‐like structures. Additionally, endothelial cells or pericytes in the diaphragmatic microvascular network may acquire α‐SMA expression through mesenchymal transformation or pericyte activation, contributing to the formation of cellular rings with contractile and profibrotic properties. Furthermore, as vascular smooth muscle cells inherently express α‐SMA, the observed increase in α‐SMA protein levels may also be partially attributed to the proliferation of these smooth muscle cells. This phenomenon may offer novel insights into the molecular mechanisms underlying MV‐induced diaphragm fibrosis. However, further experimental validation is needed to elucidate the specific mechanisms involved.

Both GsMTx4 and AAV‐mediated Piezo1 silencing effectively mitigate MV‐induced diaphragm atrophy, indicating that MV‐induced diaphragm atrophy may be partially mediated by upregulation of Piezo1. Upregulated Piezo1 triggers Ca^2+^ influx, activates calpains and inflammasomes, disrupts autophagy and impairs regeneration. Conversely, Hirata et al. demonstrated that limb immobilization down‐regulates Piezo1, lowers baseline Ca^2+^ and also causes atrophy [49]. This contradiction shows that Piezo1 plays a dual role in muscle atrophy: Both reduced expression due to insufficient mechanical stimulation and increased expression from periodic passive stretching can lead to atrophy through different pathways. These findings enhance our understanding of various types of muscle atrophy. Notably, although Nr4a1 is known to regulate skeletal muscle mass, its agonist Csn‐B did not alleviate diaphragm atrophy in this model. This suggests that Nr4a1 activation during MV may primarily exert antifibrotic effects rather than promote muscle fibre growth.

Nevertheless, this study has certain limitations. Fibrosis is a complex and heterogeneous process, and understanding its mechanisms remains challenging. Analysis of the publicly available rabbit diaphragm single‐nucleus transcriptomic dataset (GSE269980) [50] showed that 24 h of MV upregulated Piezo1 in smooth muscle cells (Log2FC = +2.02), endothelial cells (Log2FC = +0.99) and fibroblasts (Log2FC = +0.72), indicating multiple cell types' involvement in VIDD‐associated fibrosis. Lineage‐specific Piezo1 knockout models are needed to clarify Piezo1's role. Additionally, our rats were 9–13 weeks old; extending studies to fully mature and elderly animals will clarify how age modulates MV‐induced injury. Finally, besides fibrosis and atrophy, VIDD also involves disruptions in bioenergetics and excitation‐contraction coupling. Therefore, reversing this complex condition may require a strategy that combines therapies targeting multiple functional impairments.

## 5 Conclusion

The results demonstrated that with increasing duration of mechanical ventilation, the diaphragm undergoes progressive structural remodelling and functional deterioration. The mechanosensitive ion channel Piezo1 plays a pivotal regulatory role in the pathogenesis of diaphragm fibrosis induced by prolonged MV. This involvement appears to be mediated through the Akt/Nr4a1 signalling pathway. Antifibrotic interventions targeting either the inhibition of Piezo1 expression or the activation of Nr4a1 via the specific agonist Csn‐B may effectively attenuate diaphragm fibrosis and improve diaphragmatic contractile function.

## Funding

This study was supported by the Sichuan Provincial Science and Technology Plan Joint Innovation Projects (2022YFS0632).

## Conflicts of Interest

The authors declare no conflicts of interest.

## Supporting information


**Figure S1:** Representative plots of force‐frequency curves (A) and fatigue indices (B‐D).

**Figure S2:** Representative immunohistochemical images of rat diaphragm Nr4a1 and α‐SMA (400 × magnification) (A) and quantitative analysis of positive signals (B). Data are expressed as mean ± SE. One‐way ANOVA was used for all analyses, with significance denoted as follows: *p < 0.05, **p < 0.01, ***p < 0.005.

**Figure S3:** Cross‐sectional area (CSA) of rat diaphragm fibres. Data are expressed as mean±SE. Each group included five independent biological replicates. Five fields of view were randomly selected for each slice and the results were averaged. One‐way ANOVA was used for all analyses, with significance denoted as follows: *p < 0.05, **p < 0.01, ***p < 0.005.

**Table S1:** Sequences of shRNAs.
**Table S2:** Sequences of PCR primers used for amplification of target genes.
**Table S3:** List of primary antibodies used for WB, IF, and IHC.
**Table S4:** Body weight and blood gas analysis of rats in the three experimental groups.
