# Supplementary figures and images for: Mechanosensitive Piezo1 Channels Mediate Diaphragm Fibrosis Induced by Prolonged Mechanical Ventilation
Source: J Cachexia Sarcopenia Muscle. 2025 Dec 3;16(6):e70136. doi: 10.1002/jcsm.70136 (PMC12673280; doi:10.1002/jcsm.70136)

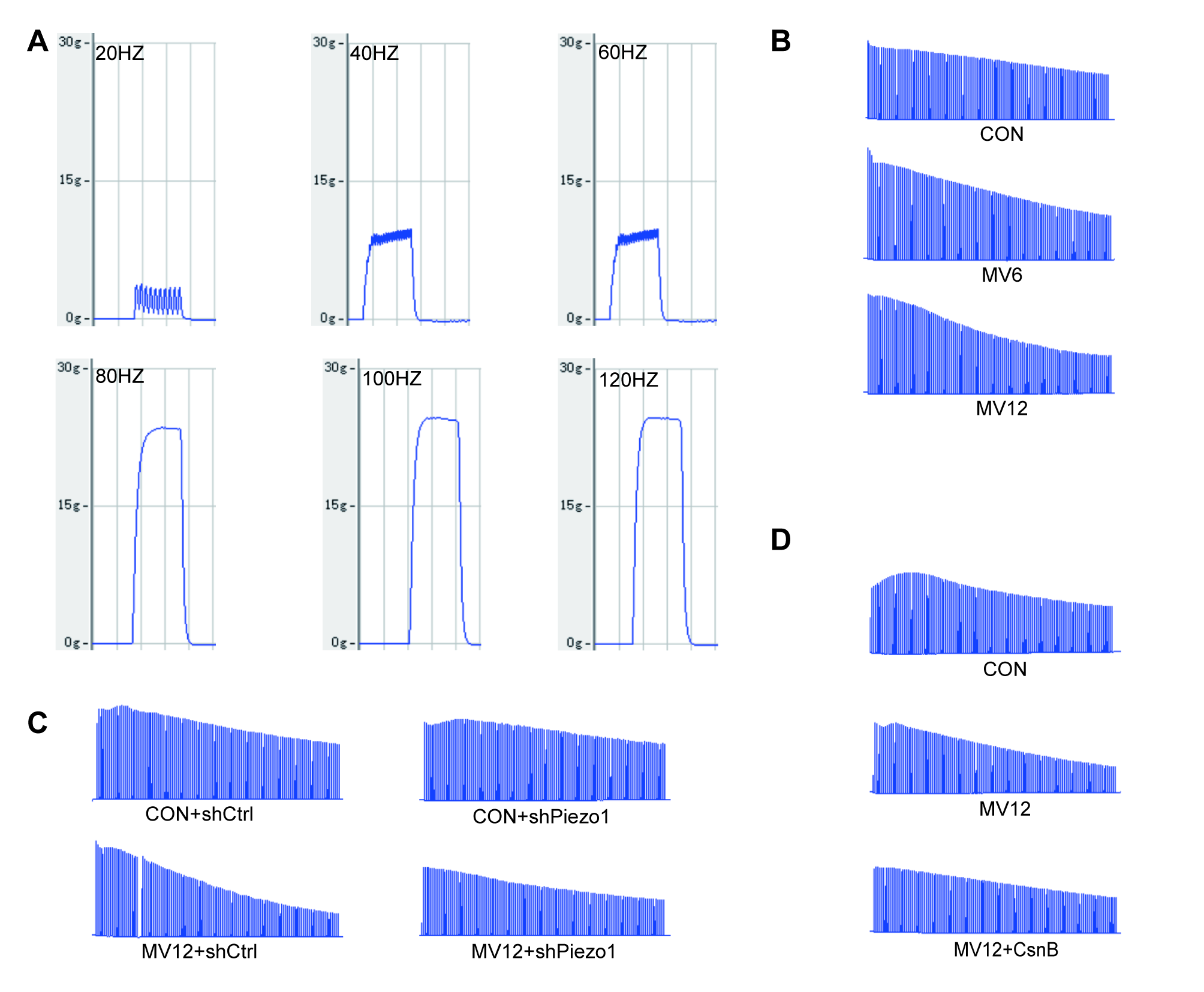

Supplement: Supplementary file 1 — Figure S1: Representative plots of force‐frequency curves (A) and fatigue indices (B‐D). [file JCSM-16-e70136-s002.tif]

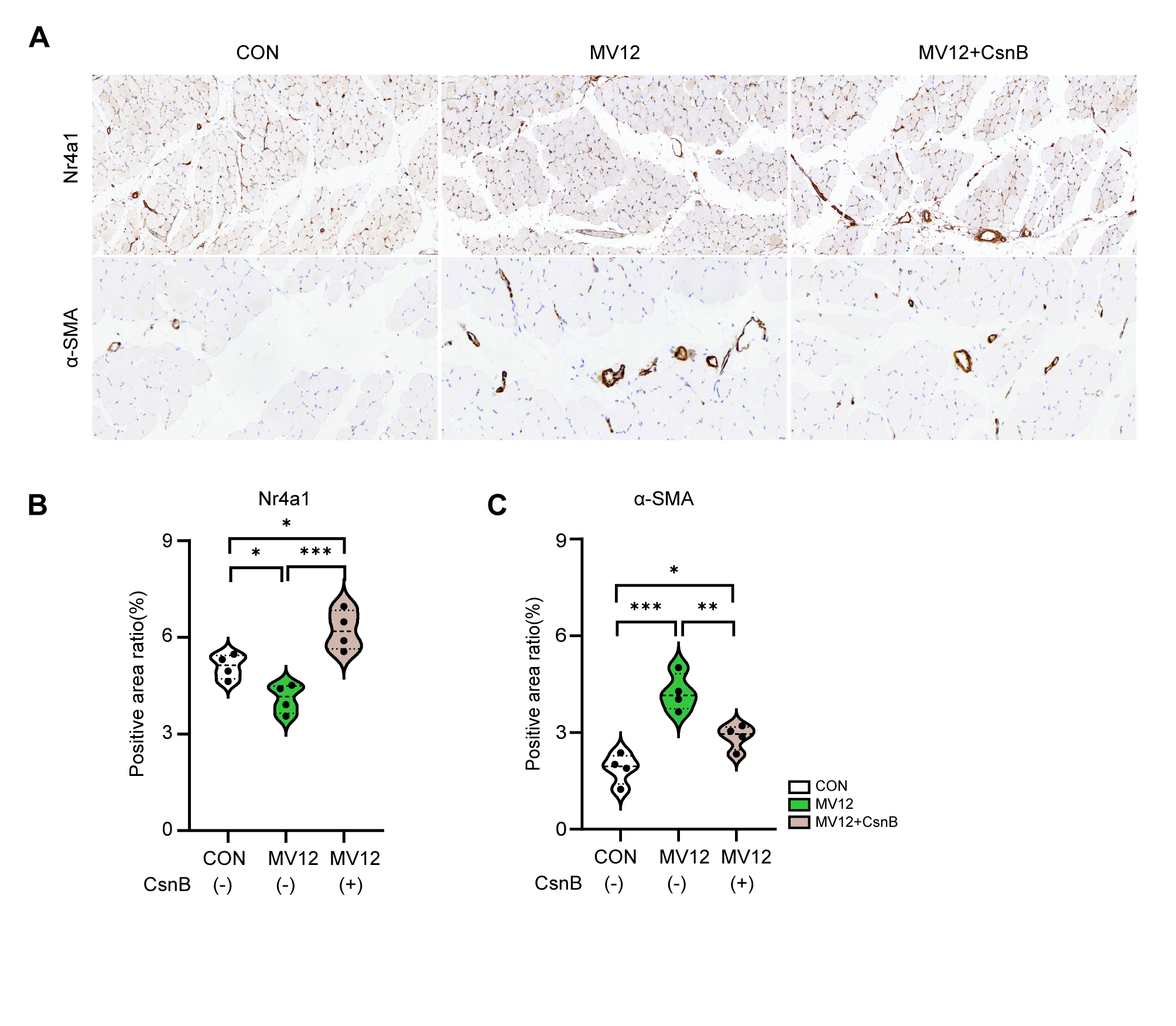

Supplement: Supplementary file 2 — Figure S2: Representative immunohistochemical images of rat diaphragm Nr4a1 and α‐SMA (400 × magnification) (A) and quantitative analysis of positive signals (B). Data are expressed as mean ± SE. One‐way ANOVA was used for all analyses, with significance denoted as follows: *p < 0.05, **p < 0.01, ***p < 0.005. [file JCSM-16-e70136-s001.tif]

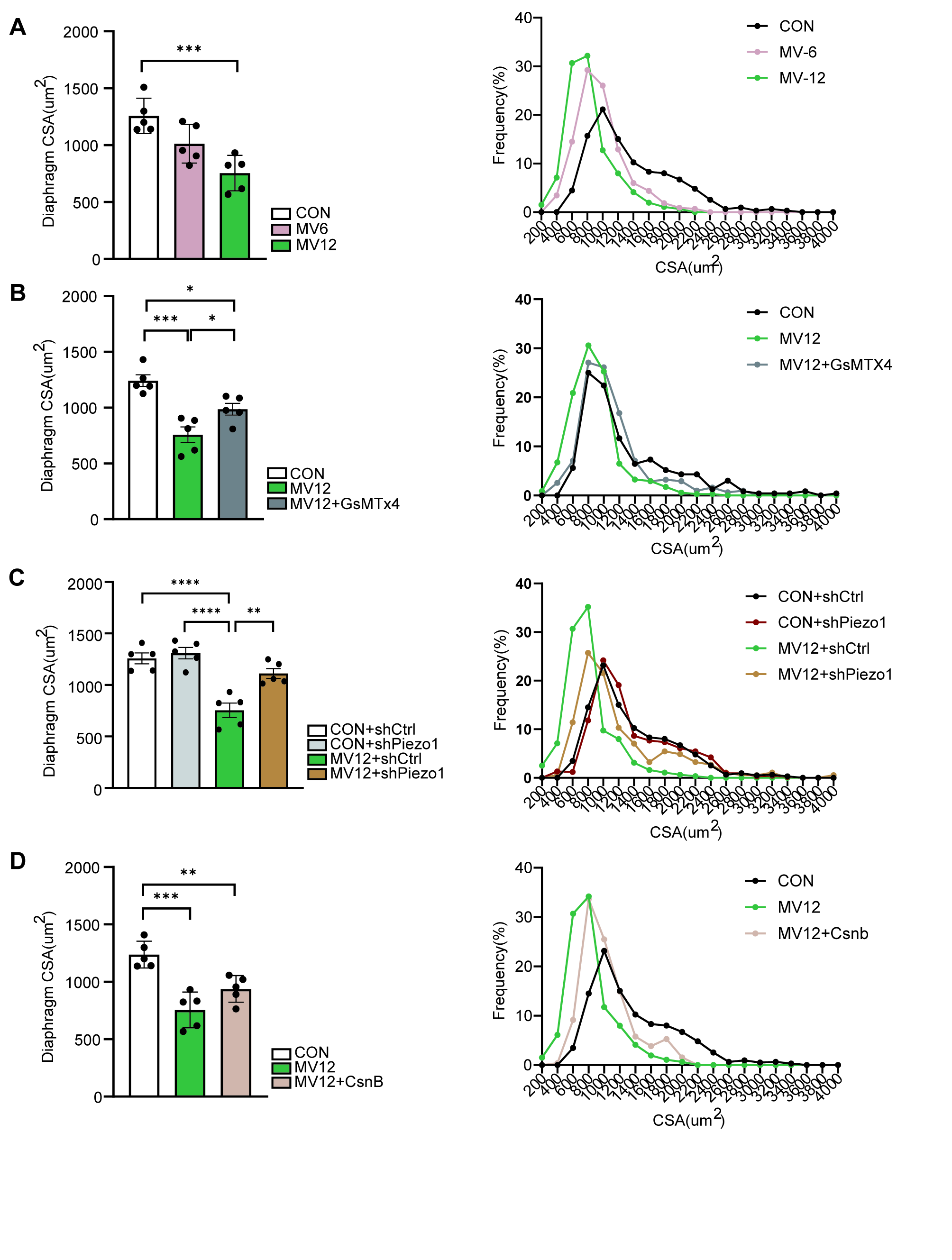

Supplement: Supplementary file 3 — Figure S3: Cross‐sectional area (CSA) of rat diaphragm fibres. Data are expressed as mean±SE. Each group included five independent biological replicates. Five fields of view were randomly selected for each slice and the results were averaged. One‐way ANOVA was used for all analyses, with significance denoted as follows: *p < 0.05, **p < 0.01, ***p < 0.005. [file JCSM-16-e70136-s003.tif]
