# Supplementary material for: Mechanosensitive Piezo1 Channels Mediate Diaphragm Fibrosis Induced by Prolonged Mechanical Ventilation
Source: J Cachexia Sarcopenia Muscle. 2025 Dec 3;16(6):e70136. doi: 10.1002/jcsm.70136 (PMC12673280; doi:10.1002/jcsm.70136)
Supplement: Supplementary file 4 — Table S1: Sequences of shRNAs. Table S2: Sequences of PCR primers used for amplification of target genes. Table S3: List of primary antibodies used for WB, IF, and IHC. Table S4: Body weight and blood gas analysis of rats in the three experimental groups. [file JCSM-16-e70136-s004.doc]

Table S1. Sequences of shRNAs

| shRNA | Target sequences (5’-3’) |
| --- | --- |
| Piezo1-shRNA-#1* | TGGGCCCTGTCAGTCTACATCAGTT |
| Piezo1-shRNA-#2 | GAGCGAAGATGAGATCGCACTGTCA |
| Piezo1-shRNA-#3 | CCAACTGGATGTGTGTGGAAGACAT |
| shRNA Ctrl | Empty vector |

* The optimal shRNA sequence that can significantly reduce the expression level of Piezo1

Table S2. Sequences of PCR primers used for amplification of target genes.

| Primers | Primers sequences |
| --- | --- |
| Piezo1 | F:CCCAACTCCACCAACCTTATCA |
|  | R:GCCACCTTCAGCATATCCAGATA |
| Piezo2 | F:CAGTGGAATGGGAAGACAGAGAA   |  | | --- | |
|  | R:GACCCAGGTGAACTTCAGGATAT |
| Trpv1 | F:GTTTCAGGGTGGACGAGGTAAAC |
|  | R:AGGGCAAAGTTCTTCCAGTTTCT |
| Trpv4 | F:TGAAGAAATGCCCTGGAGTGAAT |
|  | R:GCAAAGACCATCACAGCCAGATA |
| Trpc1 | F:CTGAGTTACCTTCGGCTCTTCTT |
|  | R:GCTCTTTGGAAGTGTACCCTTTG |
| GAPDH | F:GAAGGTCGGTGTGAACGGAT |
|  | R:CCCATTTGATGTTAGCGGGAT |

Table S3. List of primary antibodies used for WB, IF, and IHC

| Antibody | Company | Catalog No. | Dilution rate | | |
| --- | --- | --- | --- | --- | --- |
|  |  |  | WB | IF | IHC |
| Piezo1 | Proteintech | 15939-1-AP | 1:900 |  |  |
| Fibronectin | Proteintech | 15613-1-AP | 1:2000 |  |  |
| Col1a1 | HuaAn | HA722517 | 1:1000 |  |  |
| Col3 | HuaAn | HA720050 | 1:1000 |  |  |
| α-SMA | Proteintech | 14395-1-AP | 1:6000 |  |  |
| Nr4a1 | Proteintech | 12235-1-AP | 1:2000 |  | 1:500 |
| Akt1 | HuaAn | ET1609-47 | 1:2000 |  |  |
| P-Akt | HuaAn | ET1609-51 | 1:2000 |  |  |
| GAPDH | Proteintech | 60004-1-Ig | 1:6000 |  |  |
| α-SMA | Servicebio | GB13044-50 |  | 1:200 | 1：200 |

WB: western blot analysis, IHC: immunohistochemistry, IF: immunofluorescence

Table S4. Body weight and blood gas analysis of rats in the three experimental groups

|  | CON | MV6 | MV12 | P |
| --- | --- | --- | --- | --- |
| Weight(g) | 254.40±14.54 | 256.40±13.15 | 258.00±12.08 | 0.913 |
| PH | 7.39±0.05 | 7.36±0.05 | 7.35±0.03 | 0.240 |
| HCO3^-^(mmol/l) | 25.22±0.68 | 24.04±1.25 | 23.72±1.10 | 0.094 |
| PaO_2_(mmHg) | 100.06±2.38 | 98.20±1.57 | 97.28±1.67 | 0.103 |
| PaCO2(mmHg) | 39.86±3.19 | 44.16±3.84 | 45.57±3.26 | 0.055 |
| Hb(g/l) | 135.60±13.47 | 124.20±6.76 | 123.60±9.42 | 0.157 |
| Na^+^(mmol/l) | 137.90±3.28 | 135.12±1.69 | 135.67±2.11 | 0.209 |
| K^+^(mmol/l) | 3.74±0.50 | 3.68±0.29 | 3.51±0.18 | 0.584 |
| Ca^2+^(mmol/l) | 1.16±0.07 | 1.05±0.07 | 1.08±0.11 | 0.152 |
| Lac(mmol/l) | 2.00±0.18 | 2.20±0.23 | 2.30±0.17 | 0.078 |

Data are expressed as mean±SD
